# A Personalized BEST: Characterization of Latent Clinical Classes of Nonischemic Heart Failure That Predict Outcomes and Response to Bucindolol

**DOI:** 10.1371/journal.pone.0048184

**Published:** 2012-11-07

**Authors:** David P. Kao, Brandie D. Wagner, Alastair D. Robertson, Michael R. Bristow, Brian D. Lowes

**Affiliations:** 1 Division of Cardiology, University of Colorado School of Medicine, Aurora, CO; 2 Biostatistics and Informatics, Colorado School of Public Health, Aurora, CO; 3 Division of Cardiology, University of Nebraska Medical Center, Omaha, NE; University of Tampere, Finland

## Abstract

**Background:**

Heart failure patients with reduced ejection fraction (HFREF) are heterogenous, and our ability to identify patients likely to respond to therapy is limited. We present a method of identifying disease subtypes using high-dimensional clinical phenotyping and latent class analysis that may be useful in personalizing prognosis and treatment in HFREF.

**Methods:**

A total of 1121 patients with nonischemic HFREF from the β-blocker Evaluation of Survival Trial were categorized according to 27 clinical features. Latent class analysis was used to generate two latent class models, LCM A and B, to identify HFREF subtypes. LCM A consisted of features associated with HF pathogenesis, whereas LCM B consisted of markers of HF progression and severity. The Seattle Heart Failure Model (SHFM) Score was also calculated for all patients. Mortality, improvement in left ventricular ejection fraction (LVEF) defined as an increase in LVEF ≥5% and a final LVEF of 35% after 12 months, and effect of bucindolol on both outcomes were compared across HFREF subtypes. Performance of models that included a combination of LCM subtypes and SHFM scores towards predicting mortality and LVEF response was estimated and subsequently validated using leave-one-out cross-validation and data from the Multicenter Oral Carvedilol Heart Failure Assessment Trial.

**Results:**

A total of 6 subtypes were identified using LCM A and 5 subtypes using LCM B. Several subtypes resembled familiar clinical phenotypes. Prognosis, improvement in LVEF, and the effect of bucindolol treatment differed significantly between subtypes. Prediction improved with addition of both latent class models to SHFM for both 1-year mortality and LVEF response outcomes.

**Conclusions:**

The combination of high-dimensional phenotyping and latent class analysis identifies subtypes of HFREF with implications for prognosis and response to specific therapies that may provide insight into mechanisms of disease. These subtypes may facilitate development of personalized treatment plans.

## Introduction

Heart failure with reduced left ventricular ejection fraction (HFREF) develops from complex interactions between genetic factors and accumulated cardiac insults. [1] Like all heart failure patients, HFREF patients are heterogenous with respect to etiology, prognosis, and response to therapy, and our ability to identify patients likely to respond to medical therapy remains limited. In some cases, HFREF etiology directs therapy that increases the likelihood of clinical improvement. Forms of HFREF considered ‘reversible’ are often characterized by a single identifiable etiology amenable to targeted intervention. [2] There is currently no reliable way of predicting treatment response in HFREF patients who are nonischemic where a reversible etiology cannot be identified. However, normalization of LVEF in some patients with nonischemic HFREF on medical therapy in the absence of an obvious reversible etiology suggests that there may be uncharacterized reversible phenotypes.

We hypothesize that subtypes of nonischemic HFREF exist that may be differentiated by constellations of clinical features that reflect underlying pathophysiology. These subtypes may have variable clinical courses and responses to treatment, and identification of these subtypes may provide insight into mechanisms of HFREF and facilitate personalized prediction of outcomes and treatment response. Traditional outcomes-driven analyses are limited in the number of clinical features that can be evaluated due to the number of potential interactions between features contributing to the development and progression of HFREF. Latent class analysis is one statistical method of identifying groups of individuals within a population that share similar patterns of categorical variables such as symptoms or comorbid conditions, and it has been used in a number of medical disciplines including heart failure for exploration, characterization, and validation of diseases subtypes as well as for risk stratification and prediction of treatment response. [3]–[9] Latent class analysis has also been used to establish diagnostic standards for complex disease syndromes, and use of latent class analysis has been proposed as a method of dealing with large numbers of complex interactions and multiple comparisons in determining likelihood of response to interventions. [10]–[12] Briefly, latent class analysis hypothesizes the existence of unobserved classes within a population that explain patterns of association between variables and uses maximum-likelihood estimation to divide the population into subgroups by calculating a probability of subgroup membership for each symptom or comorbidity. An individual’s subgroup membership may therefore depend on the presence or absence of many different characteristics in a given model. When the population in question has a shared disease, the results are data-driven definitions of disease subtypes where each subtype is characterized by a distinct combination of clinical features. Many clinical variables can thereby be incorporated into an analytic model while preserving statistical power for outcomes analysis by identifying the most prevalent combinations of variables upon which to focus. We propose using complex phenotype descriptions of patients in combination with latent class analysis to identify subtypes of nonischemic HFREF that may have different prognoses and likelihoods of treatment response.

This is a retrospective analysis of data from the β-blocker Evaluation of Survival Trial (BEST) that generated high-dimensional phenotype descriptions of subjects using clinical data available at the time of randomization. Latent class analysis was then used to identify prevalent subtypes of HFREF, and the effect of bucindolol treatment on mortality and LVEF response was determined for each subtype. We compared the performance of our models with the Seattle Heart Failure Model (SHFM) in predicting patient mortality and LVEF improvement with bucindolol and estimated the incremental value of combining models. Models were validated by estimating unbiased area-under-the-curve c-indices within the BEST population and by applying latent class and SHFM models to an independent set of patients enrolled in the Multicenter Oral Carvedilol Heart Failure Assessment (MOCHA) Trial. [13].

## Methods

### Trial Design

The design of BEST has been described previously. [14], [15] A list of all recruitment sites is found in the Appendix S1. All patients had New York Heart Association (NYHA) class III or IV HFREF (LVEF ≤35%) and were randomized in a double-blind fashion to either bucindolol or placebo. Patients were considered ischemic if they had ≥70% obstruction in a major epicardial coronary artery by angiography or evidence of prior myocardial infarction and excluded from this analysis. [16] The primary endpoint was cumulative all-cause mortality. Secondary endpoints were all-cause mortality at one year and LVEF response defined as improvement in LVEF ≥5% with a final LVEF of ≥35% as measured using multi-gated acquisition scan (MUGA). The design of MOCHA has also been described previously. [13] All patients had an LVEF ≤35%, were mostly NYHA class II or III and had stable HF symptoms for 1 month prior to enrollment. They were randomized to placebo, low (6.25 mg bid), medium (12.5 mg bid), or high-dose (25 mg bid) carvedilol. Death and LVEF improvement as measured by MUGA were secondary endpoints in the original MOCHA analysis. Mortality data was only available up to one year of follow-up in MOCHA.

### Identification and Definition of Latent Classes

Patients were scored according to 27 clinical features (Tables 1 and 2). Criteria were encoded and applied in a MySQL server environment (Oracle Corporation, Redwood Shores, CA). [17] Patient clinical profiles were analyzed collectively using latent class analysis [18] applied to two sets of clinical variables we designated as Latent Class Models (LCM) A and B (Tables 1 and 2). LCM A and B differed only in the clinical variables included in each model. LCM A included variables that describe a patient’s non-cardiac characteristics that can contribute to the pathogenesis of HFREF including age, gender, race, body mass index, and presence of comorbidities such as diabetes, atrial fibrillation, or valvular disease. [19]–[23] LCM B included variables that describe cardiac function, progression, and severity of HFREF including right- and left-ventricular function, hemodynamic parameters such as heart rate and blood pressure, end-organ function such as estimated creatinine clearance, and signs of venous congestion such as jugular venous distension and alanine aminotransferase levels. [24]–[33] In total, 3 variables were included in both models: body mass index, creatinine clearance, and hematocrit. All 3 variables have been implicated in the pathogenesis of HFREF and can also be markers of severity of HFREF. [34], [35] They were included in both models to illustrate that the variable implications of clinical features in different contexts may be represented using this approach. [34], [36]–[40] Two sets of related variables were also included: age of HF onset (LCM A) vs. chronologic age (LCM B) and presence of hypertension (LCM A) vs. presence of hypotension (LCM B). Age of HF onset, a static value, may be relevant to the HFREF etiology, while chronologic age may be related to HF progression. Similarly, presence of hypertension (LCM A) may be related to HF etiology while hypotension (LCM B) may be a marker of advanced HF.

**Table 1:** Features of HFREF Latent Class Model A subtypes.

	A1	A2	A3	A4	A5	A6	All
	18.6% (208)	14.4% (161)	16.6% (186)	14.5% (162)	7.8% (87)	28.3% (317)	n = 1121
Age of HF onset, years							
<30	0.0%	3.7%	0.0%	35.8%	1.1%	1.6%	6.2%
30–45	13.9%	25.5%	17.7%	56.2%	11.5%	29.7%	26.6%
45–60	38.9%	38.5%	52.2%	8.0%	37.9%	50.2%	39.7%
>60	47.1%	32.3%	30.1%	0.0%	49.4%	18.6%	27.5%
Male	83.7%	39.1%	30.1%	47.5%	79.3%	100.0%	67.4%
Race							
White, non-Hispanic	36.1%	42.2%	96.2%	34.0%	87.4%	68.8%	59.9%
Black, non-Hispanic	54.8%	47.2%	1.1%	55.6%	5.7%	23.0%	32.1%
Hispanic	7.7%	8.7%	2.7%	10.5%	2.3%	5.4%	6.3%
Asian/Pacific Islander	1.4%	0.6%	0.0%	0.0%	2.3%	1.3%	0.9%
American Indian	0.0%	1.2%	0.0%	0.0%	0.0%	1.3%	0.5%
Other	0.0%	0.0%	0.0%	0.0%	2.3%	0.3%	0.3%
Body Mass Index, kg/m^2^							
<18.5	7.7%	0.0%	2.7%	0.0%	2.3%	0.0%	2.1%
18.5–25	63.9%	17.4%	33.3%	27.2%	50.6%	10.7%	30.7%
25–30	19.2%	31.7%	31.2%	23.5%	37.9%	36.9%	30.1%
>30	9.1%	50.9%	32.8%	49.4%	8.0%	52.4%	37.0%
Diabetes Mellitus							
None	73.1%	25.5%	88.2%	92.6%	94.3%	67.2%	71.5%
Present	21.6%	51.6%	7.0%	6.8%	3.4%	23.7%	20.5%
Present + end-organ damage	5.3%	23.0%	4.8%	0.6%	2.3%	9.1%	7.9%
Blood pressure, mm Hg							
<120/80	15.9%	0.0%	14.0%	19.1%	31.0%	0.6%	10.6%
120–140/80–90	10.1%	5.0%	44.6%	22.2%	34.5%	18.0%	21.0%
140–160/90–100	65.4%	57.8%	39.8%	41.4%	27.6%	59.0%	51.8%
>160/100	8.7%	37.3%	1.6%	17.3%	6.9%	22.4%	16.6%
Total cholesterol, mg/dL							
<200	65.4%	6.2%	9.7%	61.7%	42.5%	24.6%	33.8%
200–240	18.8%	15.5%	28.5%	26.5%	29.9%	22.4%	22.9%
>240	15.9%	78.3%	61.8%	11.7%	27.6%	53.0%	43.3%
Triglycerides, mg/dL							
<150	91.7%	8.9%	10.9%	48.4%	40.2%	2.3%	30.2%
150–250	8.3%	20.9%	33.7%	37.1%	32.9%	39.5%	28.4%
>250	0.0%	70.3%	55.4%	14.5%	26.8%	58.2%	38.7%
Creat. Cl., ml/min*1.73 m^2^							
>90	2.9%	3.7%	9.1%	34.0%	5.7%	14.2%	12.0%
60–90	37.0%	24.2%	41.9%	52.5%	25.3%	52.7%	41.7%
30–60	49.5%	53.4%	45.2%	13.6%	59.8%	30.6%	39.6%
15–30	9.6%	15.5%	3.8%	0.0%	9.2%	2.5%	6.1%
<15	1.0%	3.1%	0.0%	0.0%	0.0%	0.0%	0.6%
Hematocrit, %							
>40	4.8%	0.0%	0.0%	0.0%	5.7%	11.4%	4.5%
30–40	57.2%	28.6%	45.7%	45.7%	58.6%	88.6%	58.5%
20–30	35.1%	69.6%	54.3%	52.5%	35.6%	0.0%	35.9%
<20	2.9%	1.9%	0.0%	1.9%	0.0%	0.0%	1.1%
Atrial fibrillation	24.5%	9.3%	8.1%	6.2%	86.2%	22.1%	21.1%
Left bundle branch block	23.1%	19.9%	67.7%	7.4%	10.3%	16.1%	24.8%
Pacemaker	6.7%	0.0%	0.5%	4.3%	42.5%	3.5%	6.2%
Mitral valve disease	1.9%	0.0%	3.2%	1.2%	48.3%	2.2%	5.4%
Aortic valve disease	3.8%	0.0%	1.1%	0.0%	21.8%	1.3%	2.9%
History of sudden cardiac death	2.9%	4.3%	5.4%	2.5%	16.1%	2.5%	4.4%

**Table 2:** Features of HFREF Latent Class Model B subtypes.

	B1	B2	B3	B4	B5	All subjects
	22.6% (253)	33.8% (379)	23.0% (258)	11.7% (131)	8.9% (100)	n = 1121
Age, years						
<30	0.0%	3.2%	0.0%	22.1%	7.0%	4.3%
30–45	0.4%	29.8%	6.2%	53.4%	21.0%	19.7%
45–60	24.9%	49.9%	43.4%	23.7%	38.0%	38.6%
>60	74.7%	17.2%	50.4%	0.8%	34.0%	37.4%
LVEF, %						
>55	0.4%	0.0%	0.0%	0.0%	0.0%	0.1%
45–55	0.0%	0.0%	0.0%	0.0%	0.0%	0.0%
35–45	0.8%	0.3%	0.0%	0.0%	0.0%	0.3%
25–35%	60.1%	48.3%	19.8%	35.1%	15.0%	39.9%
<25%	38.7%	51.5%	80.2%	64.9%	85.0%	59.8%
RVEF, %						
>55%	21.9%	6.6%	6.3%	5.8%	0.0%	7.4%
45–55%	20.4%	19.0%	9.5%	11.7%	8.3%	12.3%
35–45%	28.1%	29.8%	18.0%	26.2%	22.6%	20.7%
25–35%	18.4%	26.9%	32.0%	22.3%	13.1%	19.9%
<25%	11.2%	17.7%	34.2%	34.0%	56.0%	20.9%
QRS, msec						
<120	36.0%	77.3%	45.0%	76.3%	56.0%	58.5%
120–150	24.5%	5.5%	19.0%	8.4%	23.0%	14.8%
>150	39.5%	17.2%	36.0%	15.3%	21.0%	26.7%
Heart rate, bpm						
<60	13.4%	5.3%	5.0%	4.6%	6.0%	7.0%
60–80	55.7%	35.6%	36.8%	22.1%	18.0%	37.3%
80–100	29.6%	42.2%	50.8%	46.6%	49.0%	42.5%
100–120	1.2%	15.6%	7.0%	19.8%	24.0%	11.6%
>120	0.0%	1.3%	0.4%	6.9%	3.0%	1.6%
Systolic blood pressure, mm Hg						
>120	70.8%	63.6%	6.2%	0.0%	22.0%	41.7%
110–120	20.9%	28.2%	14.3%	2.3%	10.0%	18.7%
100–110	7.1%	5.3%	31.4%	44.3%	20.0%	17.6%
90–110	1.2%	0.3%	31.4%	42.7%	26.0%	14.9%
<90	0.0%	0.0%	16.7%	10.7%	22.0%	7.0%
Pulse pressure, mm HG						
>40	87.0%	65.7%	7.8%	12.2%	26.0%	47.4%
25–40	6.3%	30.9%	79.5%	74.0%	49.0%	43.2%
<25	0.0%	1.1%	12.8%	13.7%	23.0%	7.0%
Jugular venous distension						
Not present	60.1%	63.3%	51.2%	58.0%	26.0%	55.8%
Base of neck	27.7%	23.7%	31.0%	20.6%	22.0%	25.8%
Halfway up	8.7%	10.6%	14.0%	15.3%	35.0%	13.6%
Angle of mandible	3.2%	2.4%	3.9%	6.1%	17.0%	4.6%
Blood Urea Nitrogen, mg/dL						
<10	1.6%	17.4%	2.3%	16.8%	0.0%	8.7%
10–25	67.2%	77.8%	67.4%	79.4%	3.0%	66.5%
25–40	19.8%	4.7%	25.6%	0.8%	38.0%	15.4%
40–55	7.5%	0.0%	4.7%	3.1%	23.0%	5.2%
>55	4.0%	0.0%	0.0%	0.0%	35.0%	4.0%
Alanine aminotransferase, U/L						
<25	79.4%	45.1%	60.9%	37.4%	53.0%	56.0%
25–50	19.4%	44.9%	31.0%	53.4%	25.0%	35.1%
50–75	1.2%	7.4%	7.0%	8.4%	14.0%	6.6%
>75	0.0%	2.4%	0.8%	3.1%	8.0%	2.1%
Serum sodium, mEq/L						
>140	38.9%	34.7%	29.6%	16.9%	9.1%	29.5%
130–140	59.8%	65.0%	69.6%	80.0%	83.8%	67.3%
<130	1.3%	0.3%	0.8%	3.1%	7.1%	1.5%
Body Mass Index, kg/m^2^						
<18.5	3.6%	0.5%	4.3%	0.8%	0.0%	2.1%
18.5–25	41.1%	14.5%	43.8%	29.0%	35.0%	30.7%
25–30	39.5%	24.0%	32.9%	32.8%	18.0%	30.1%
>30	15.8%	60.9%	18.6%	37.4%	47.0%	37.0%
Creat. Clearance, ml/min*1.73 m^2^						
>90	1.2%	20.8%	0.0%	39.7%	0.0%	12.0%
60–90	26.5%	61.5%	34.9%	56.5%	4.0%	41.7%
30–60	59.7%	17.2%	65.1%	3.1%	56.0%	39.6%
15–30	11.1%	0.5%	0.0%	0.0%	38.0%	6.1%
<15	1.6%	0.0%	0.0%	0.8%	2.0%	0.6%
Hematocrit, %						
>40	1.2%	2.9%	6.6%	6.9%	11.0%	4.5%
30–40	43.1%	72.6%	63.6%	49.6%	43.0%	58.5%
20–30	54.5%	24.5%	29.5%	40.5%	42.0%	35.9%
<20	1.2%	0.0%	0.4%	3.1%	4.0%	1.1%

Latent class analysis was performed using the *poLCA* function in the R statistical package. [[NO STYLE for: Linzer 2010],[NO STYLE for: Team 2010]] The optimal clinical profiles according to the variables in each latent class model were derived in the form of subtype-conditional probabilities for each variable for a range of 2–10 subtypes. Error statistics were calculated for each model iteratively to determine the optimum number of latent classes. The number of latent classes corresponding to the first local χ^2^ minimum following the first minimum of the Bayesian information criterion was selected, and the corresponding model was used for all subsequent analyses. [[NO STYLE for: Linzer 2010]] The most likely LCM A and LCM B subtype were determined for each patient in a Bayesian fashion (See Appendix S1), and descriptive statistics were compiled. SHFM Score and corresponding predicted mortality at one year were calculated for all patients both with and without the SHFM β-blocker coefficient. [43] The SHFM Score including the β-blocker coefficient was used to assess overall performance of SHFM Score in the BEST population, and the SHFM Score excluding the β-blocker coefficient was used for all analyses investigating the treatment effect of bucindolol. All multivariate predictors in the SHFM were available in the BEST trial with the exception of percent lymphocytes, and a value of 25% was imputed for all patients based on the validation sets for SHFM. [43].

### Association between Latent Class Models and Outcomes

Cox proportional-hazards models and the log-rank test were used to examine the associations between latent classes and cumulative all-cause mortality according to the intention-to-treat principle. These models were fit using the coxph and survfit functions from the *survival* library in the R statistical package. [[NO STYLE for: Therneau 2010]] Logistic regression models were used for the one-year mortality and LVEF response outcomes. Interactions between latent classes and the treatment groups were used to estimate the response to treatment within each subtype. For survival models, an interaction with time was included for those variables that did not meet the proportional hazards assumption.

Multivariate models comprised of all possible combinations of LCM A, B, and SHFM Score were generated to identify those that provide the best discrimination between outcome variables. Cox proportional hazards models and the log-rank test were used to study discrimination of all-cause mortality, whereas logistic regression was used to study discrimination of one-year mortality and LVEF response. All models included treatment group as a covariate, and c-indices were calculated for model comparison. [45,[NO STYLE for: Liu 2009]] Improvement in risk prediction with the addition of LCM A and B to SHFM Score according to logistic regression was assessed by comparing c-indices with the χ^2^ test and by calculation of net reclassification improvement measures (NRI). [47] Logistic regression, NRI, and ROC calculations were performed using SAS version 9.2 software. (SAS Institute Inc., Cary, NC, 2008).

### Validation of Multivariate Models

Performance of the Cox and logistic regression models in predicting all-cause mortality, one-year mortality and LVEF response was further validated by estimating unbiased c-indices for both outcomes using leave-one-out cross-validation in the BEST dataset and by applying the estimates from the predictive models calculated on the BEST population to nonischemic HFREF patients from the independent MOCHA population. [13].

## Results

### Patient Characteristics

In all, 1121/2708 patients enrolled in BEST were identified as nonischemic and included in all subsequent analyses. A total of 6 LCM A and 5 LCM B subtypes were identified. Distributions of clinical variables for all subjects according to subtype are shown in Tables 1 and 2, respectively. The subtype-conditional probabilities for each explanatory variable used to calculate an individual’s LCM A and LCM B subtype are given in Tables S1 and S2 of the Appendix S1 along with an illustration of how to calculate a patient’s subtype.

### Latent Class Model A (Table 1)

LCM A subtypes were characterized by distinct collections of clinical features that frequently resembled known HFREF syndromes. Subtype A1 was characterized by advanced age of onset, non-Caucasian race, male gender, HTN, mild-moderate renal insufficiency, and elevated rates of atrial fibrillation (24.5%). Subtype A2 was characterized by middle age of onset, female gender, moderate renal insufficiency, anemia, high body mass index, and very high rates of diabetes mellitus (74.6%), hypertension (95.0%), hyperlipidemia (93.8%), and hypertriglyceridemia (91.1%). Subtype A3 was characterized by middle age of onset, female gender, Caucasian race, hyperlipidemia, hypertriglyceridemia, anemia, and the presence of left bundle branch block (LBBB). Subtype A4 was characterized by young age of onset, non-Caucasian race, obesity, anemia, and lower rates of traditional cardiac risk factors such as hyperlipidemia, hypertriglyceridemia, and diabetes mellitus. Subtype A5 was characterized by advanced age of onset, Caucasian race, atrial fibrillation (86.2%), mitral valve disease (48.3%), aortic valve disease (21.8%), history of pacemaker placement (42.5%), and a significantly higher rate of prior sudden cardiac death (16.1%). This subtype had the smallest number of subjects (7.8%), whereas subtype A6 was the largest with 28.3% of subjects. Subtype A6 was characterized by middle age of onset, Caucasian race, male gender (100%), high body mass index, hypertension, hyperlipidemia, and hypertriglyceridemia with less associated diabetes mellitus (32.8%) than was seen in Subtype A2.

### Latent Class Model B (Table 2)

Subjects were fairly evenly divided among LCM B subtypes with subtypes B3 and B5 having slightly smaller percentages of 11.7% and 8.9%, respectively. Subtypes B1–B3 were characterized by preserved systolic blood pressure, pulse pressure, RVEF, and renal function with few signs of volume overload such as jugular venous distention and elevated serum alanine aminotransferase. Ventricular function declined first followed by worsening of hemodynamic parameters and finally by signs of venous congestion. In subtypes B4 and B5, right ventricular ejection fraction, systolic and pulse pressure were lower, heart rate was higher, renal function and hyponatremia were worse, and jugular venous distension, blood urea nitrogen, and serum alanine aminotransferase were higher. Examination of features such as age and body mass index suggest that while many of the clinical features are markers of heart failure severity, LCM B subtypes may not represent a continuous progression of illness shared among all HFREF patients in this study.

### Association with Outcomes

In LCM A, subtype A1 had the highest event rates with 43.8% cumulative all-cause mortality, 18.3% one-year mortality, and an LVEF response rate of only 14.4%, whereas subtypes A2 and A6 had some much lower event rates with cumulative all-cause morality of 16.8% and 18.9%, respectively, and LVEF response rates of 31.1% and 36.0%, respectively. A much larger range in cumulative mortality rates were observed for the LCM B subtypes; subtype B5 had the highest overall mortality rate at 54.0% (62.2% for placebo -treated patients), while subtype B1 had the lowest at 15.3% (17.5% for placebo-treated patients). These ranks were consistent across one-year mortality and lack of LVEF response.

The predicted one-year mortality for all patients using SHFM was 11.0% compared with an observed one-year mortality of 9.6%. The hazard ratio (HR) and corresponding confidence interval (CI) for cumulative all-cause mortality associated with each unit increase in SHFM Score including the β-blocker coefficient was 2.92 (95% CI 2.40–3.54, p<0.0001). The c-index for SHFM Score including the β-blocker coefficient was 0.76 (95% CI 0.66–0.85), which was comparable to all SHFM validation sets (range 0.60–0.81). [43]. The likelihood of LVEF response decreased significantly with each unit increase in SHFM Score (OR 0.29, 95% CI 0.22–0.38), and SHFM performed well in predicting LVEF response (c-index = 0. 68).

### Differences in Treatment Effects Between Latent Classes

**Figure 1:**
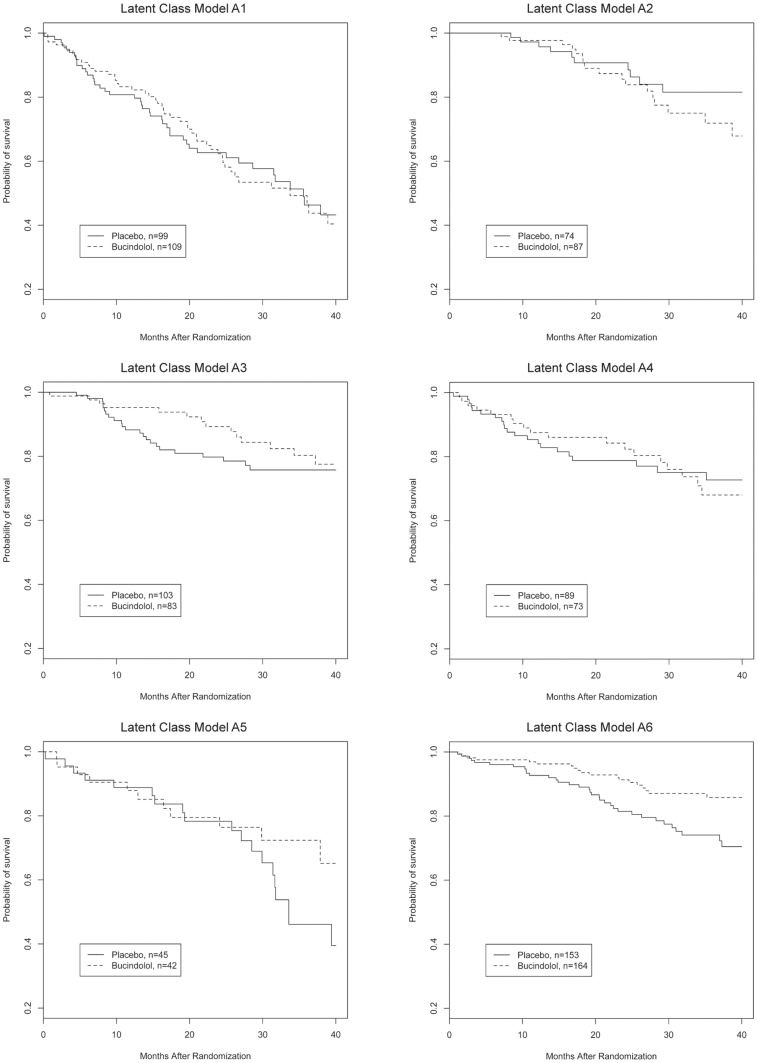
Kaplan-Meier survival curves according to Latent Class Model A subtype, bucindolol vs. placebo.

**Figure 2:**
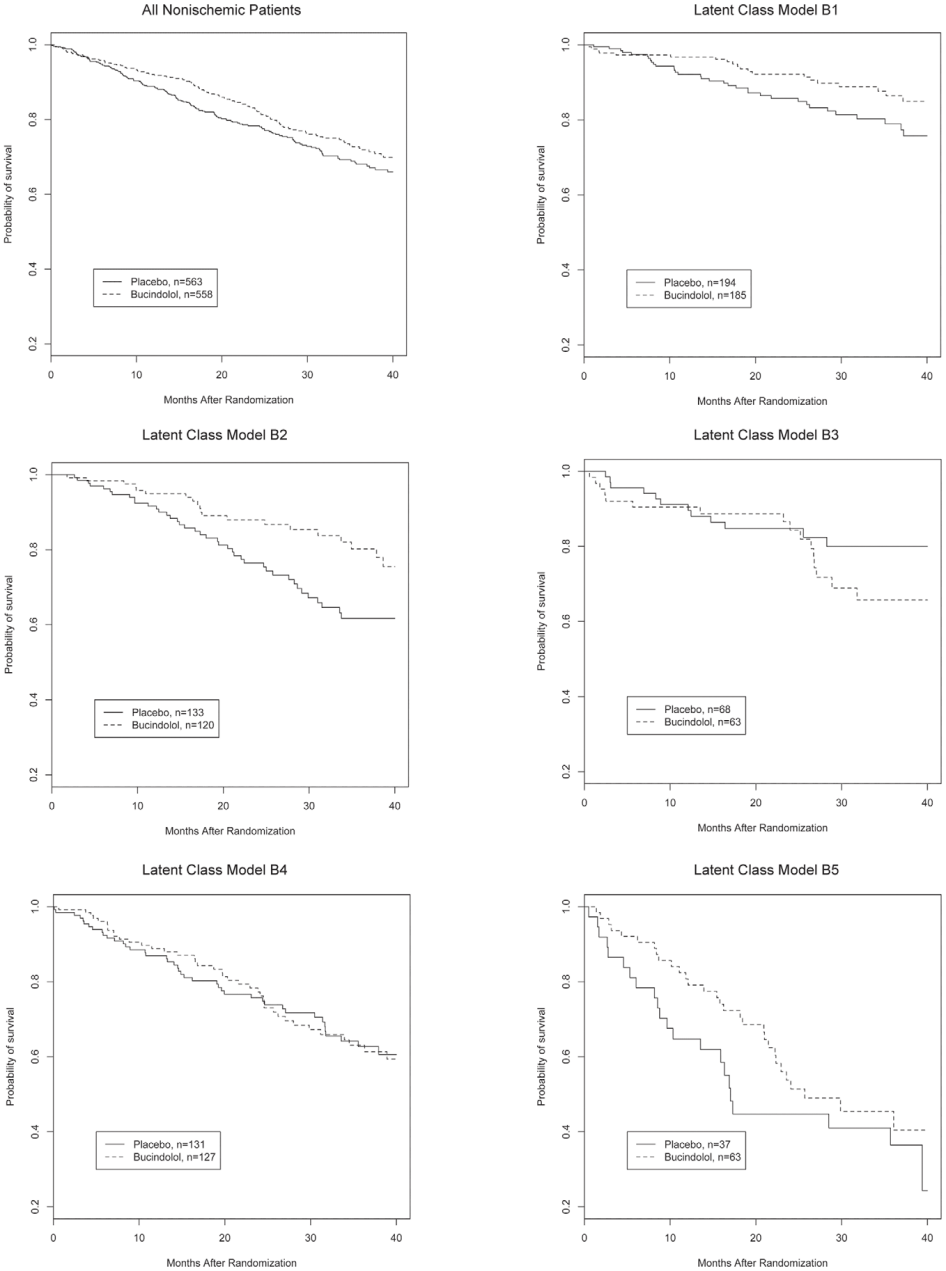
Kaplan-Meier survival curves for all nonischemic patients and according to Latent Class Model B subtype, bucindolol vs. placebo.

A total of 151/563 patients (26.8%) in the placebo group and 131/558 (23.5%) patients in the bucindolol group died (HR 0.82, 95% CI 0.65–1.04, p = 0.1). Response to treatment was evaluated for all three outcomes within each subtype (Table 3). Response to bucindolol as measured by cumulative survival varied significantly in both LCM A and B models. Subtype A1 showed no reduction in cumulative all-cause mortality associated with bucindolol, whereas subtype A6 showed an absolute and relative risk reduction of 10.2% and 42% respectively (Figure 1, p = 0.01). In LCM B, only subtype B2 showed significant improvement in mortality associated with bucindolol (Figure 2, p = 0.01). As these figures indicate, there was a time-varying effect of treatment in LCM A2 and A4. Therefore, the HRs presented in Table 3 are average HRs over the observed death times. To further assess the effect of treatment within each LCM subtype at a single clinically meaningful time-point, the model using one-year mortality was also characterized fully.

**Table 3:** Outcomes according to subtype.

	Total Number		Cumulative mortality, all-cause				One year mortality, all-cause				LVEF response			
LCM A	Plac.	Buc.	Plac,%	Buc,%	Hazard ratio (95% CI)	p	Plac,%	Buc,%	Odds ratio(95% CI)	p	Plac,%	Buc,%	OR (95% CI)	p
A1	99	109	44.4	43.1	0.97 (0.64–1.46)	0.88	19.2	17.4	0.89 (0.44–1.80)	0.74	15.2	13.8	0.89 (0.41–1.95)	0.76
A2	74	87	13.5	19.5	1.45 (0.68–3.20)	0.35	2.7	2.3	0.85 (0.12–6.20)	0.87	23.0	37.9	2.05 (1.03–4.17)	0.04
A3	103	83	22.3	18.1	0.77 (0.40–1.48)	0.43	11.7	4.8	0.38(0.12–1.24)	0.11	15.5	39.8	3.59 (1.82–7.31)	0.0003
A4	89	73	23.6	24.7	0.99 (0.53–1.86)	0.99	15.7	12.3	0.75 (0.31–1.83)	0.54	15.7	27.4	2.02 (0.93–4.43)	0.07
A5	45	42	42.2	26.2	0.61 (0.29–1.28)	0.18	11.1	11.9	1.08 (0.28–4.04)	0.91	8.9	28.6	4.10 (1.29–15.82)	0.02
A6	153	164	24.2	14.0	0.52 (0.31–0.88)	0.01	7.2	3.7	0.49 (0.18–1.36)	0.17	25.5	45.7	2.46 (1.54–3.99)	0.0002
LCM B														
B1	194	185	17.5	13.0	0.67 (0.40–1.13)	0.13	7.7	3.2	0.40 (0.14–1.01)	0.06	25.8	48.6	2.72 (1.79–4.22)	<0.0001
B2	133	120	30.1	16.7	0.55 (0.30–0.86)	0.01	8.3	5.0	0.58 (0.20–1.59)	0.30	18.0	38.3	2.82 (1.60–5.08)	0.0004
B3	68	63	19.1	25.4	1.42 (0.68–2.94)	0.37	10.3	9.5	0.92 (0.28–2.92)	0.88	16.2	30.2	2.24 (0.98–5.32)	0.06
B4	131	127	33.6	31.5	0.94 (0.61–1.44)	0.78	13.0	11.0	0.83 (0.39–1.76)	0.63	14.5	18.1	1.30 (0.67–2.55)	0.43
B5	37	63	62.2	49.2	0.69 (0.40–1.18)	0.12	35.1	20.6	0.58 (0.19–1.19)	0.11	2.7	15.9	6.79 (1.22–127.37)	0.07
All	563	558	26.8	23.5	0.82 (0.65–1.04)	0.10	11.2	8.1	0.70 (0.46–1.04)	0.08	18.7	33.7	2.22 (1.69–2.93)	<0.0001

Plac = placebo, Buc = bucindolol.

The effect of bucindolol on one-year mortality trended towards benefit for those in the A6 and B2 classes, but did not reach statistical significance for either class. There was a marginally significant difference for A3 at 12 months, but this difference disappeared at subsequent follow-up (Figure 1). Bucindolol was associated with a significant increase in likelihood of LVEF response in subtypes A2, A3, A5, and A6 and ranged from no effect in subtype A1 to a 156% relative and 24.3% absolute increase in subtype A3. The likelihood of improvement in LVEF increased comparably across all B subtypes both in relative and absolute terms but only reached statistical significance in subtypes B1 and B2.

### Combined Models

Multivariate survival and logistic regression models were then constructed to determine whether LCM A and B classification added predictive information to each other and to the SHFM. Multivariate Cox hazard ratios for cumulative mortality are shown in Table 4. Consistent with the descriptive event rates presented earlier, subtype A1 had the highest cumulative mortality even after adjusting for LCM B and SHFM Score, and subtypes A2 and A6 had survival rates 50–70% better than those in A1. Significant time interactions where observed for subtypes A2 and A4. The HRs comparing the risk of subtype A2 with A1 increased over time, with HR estimates from the full model at 1, 2, and 3 years of 0.32 (95% CI 0.19–0.52), 0.51 (95% CI 0.31–0.82) and 0.67 (95% CI 0.34–1.14), respectively. In contrast, the HR comparing the risk of subtype A4 decreased over time with a HR at 1, 2, and 3 years of 0.69 (95% CI 0.44–1.08), 0.55 (95% CI 0.33–0.94), and 0.49 (95% CI 0.27–0.89), respectively. When LCM B and SHFM Score were combined, the risk for subjects in subtype B5 remained a significantly different from subjects in B1 (HR 2.12, 95% CI 1.35–3.34). When combined with SHFM Score, all subtypes except for A5 had a lower mortality compared to subtype A1. Both LCM A and B remained significant predictors of mortality after adjusting for risk associated with treatment and SHFM Score (p<0.01).

**Table 4:** Cox proportional hazards ratio, cumulative mortality for individual and combined models.

	Cox Multivariate HR – Cumulative all-cause mortality						
Bucindolol	0.82(0.65–1.04)	0.76(0.60–0.96)	0.77(0.61–0.97)	0.77(0.61–0.98)	0.78(0.62–0.99)	0.75(0.60–0.95)	0.76(0.60–0.96)
A1	Reference	–	–	Reference	Reference	–	Reference
A2* Time int	0.39(0.25–0.61) 1.92(1.04–3.51)	–	–	0.34(0.22–0.52)	0.50(0.32–0.79)2.00(1.07–3.74)	–	0.48(0.30–0.75) 1.98(1.06–3.68)
A3	0.34(0.23–0.50)	–	–	0.40(0.27–0.58)	0.48(0.33–0.71)	–	0.49(0.33–0.72)
A4* Time int	0.35(0.22–0.56) 0.72(0.53–0.97)	–	–	0.63(0.41–0.98)	0.48(0.30–0.76)0.73(0.54–0.98)	–	0.57(0.34–0.95) 0.73(0.54–0.99)
A5	0.65(0.43–0.98)	–	–	0.64(0.42–0.97)	0.67(0.44–1.02)	–	0.65(0.43–0.99)
A6	0.32(0.23–0.44)	–	–	0.41(0.29–0.59)	0.47(0.33–0.66)	–	0.49(0.35–0.70)
B1	–	Reference	–	Reference	–	Reference	Reference
B2	–	0.63(0.44–0.90)	–	0.69(0.47–1.02)	–	0.75(0.52–1.08)	0.79(0.53–1.17)
B3	–	1.41(1.01–1.96)	–	1.28(0.92–1.79)	–	0.97(0.69–1.37)	0.92(0.65–1.31)
B4	–	0.93(0.60–1.45)	–	0.94(0.57–1.54)	–	0.76(0.49–1.19)	0.76(0.46–1.26)
B5	–	3.25(2.24–4.71)	–	3.02(2.08–4.40)	–	1.58(1.04–2.42)	1.64(1.07–2.51)
SHFM Score	–	–	3.06(2.50–3.73)	–	2.70(2.17–3.35)	2.47(1.93–3.16)	2.26(1.74–2.93)
Wald χ^2^	77.5(p<.0001)	83.3(p<.0001)	121.58(p<.0001)	138.7(p<.0001)	156.5(p<.0001)	141.0(p<.0001)	176.6(p<.0001)

* estimates represent the HR at the geometric mean of the survival times, approximately 1.8 years.

**Figure 3:**
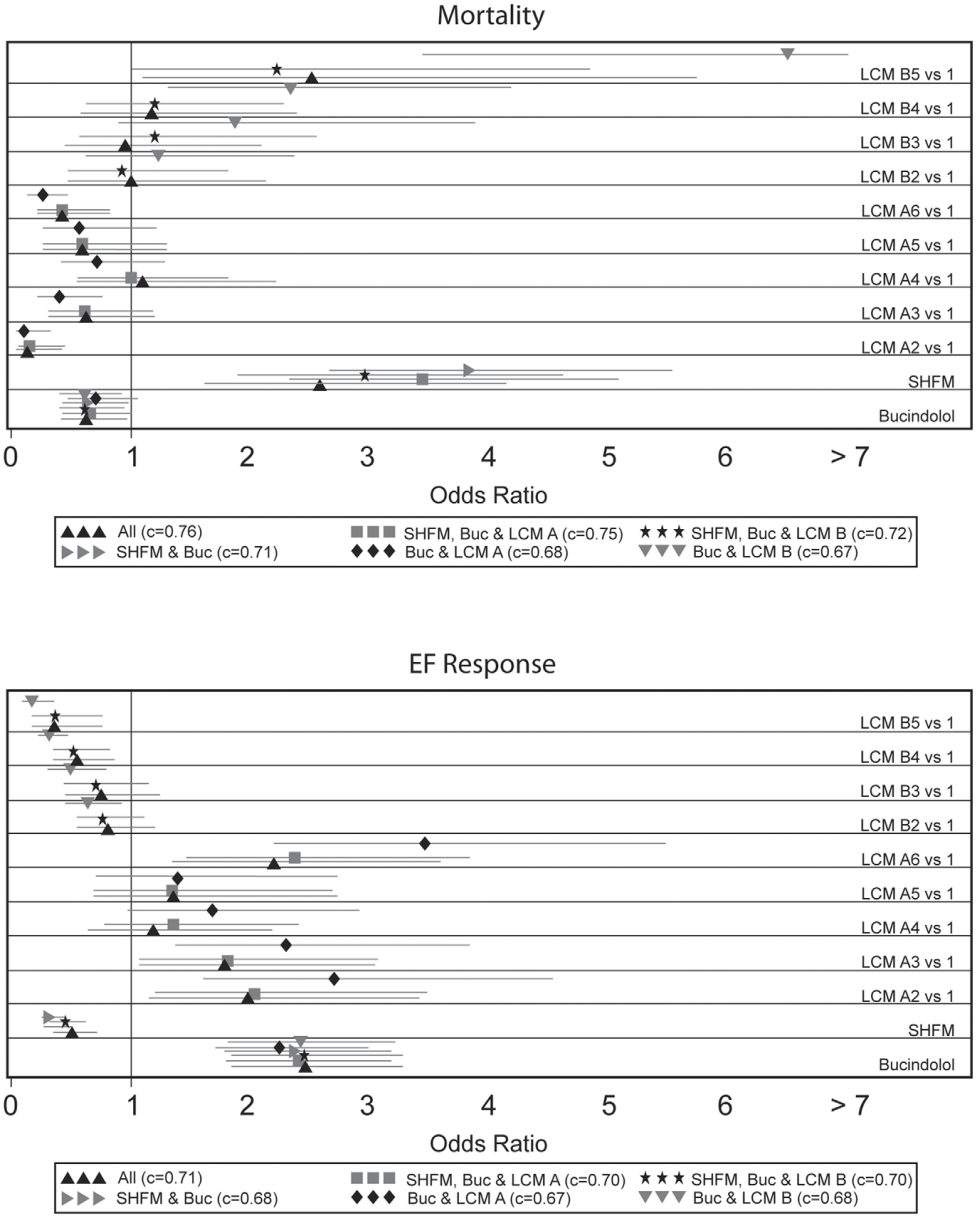
Logistic regression odds ratio for mortality and EF response according to Latent Class Models A and B subtype, SHFM Score and bucindolol treatment.

Results of multivariate logistic regression are shown in Figure 3. LCM A and B were highly significant for predicting both one-year mortality and LVEF response for both models (p<0.01). LCM A remained highly significant in predicting both mortality and LVEF response when combined with SHFM Score. LCM B remained significant in predicting LVEF response in combination with SHFM Score, but did not remain significant in predicting mortality. Membership in subtype B5 remained an independent predictor of mortality with a hazard ratio of 2.23 (95% CI 1.02–4.85) relative to subtype B1. When all three factors were included, LCM A and SHFM Score and subtype B5 remained significant predictors of one-year mortality. LCM A, B, and SHFM Score were all multivariate predictors of LVEF response.

### Model Comparisons

Comparisons of all 7 combinations of predictors displayed in Table 4 were made for the three outcome measures (cumulative mortality, one-year mortality and LVEF response) using c-indices (Table 5). [43] The model which included LCM A alone appeared to have the best predictive ability for cumulative mortality overall. Models with LCM A, B and their combination performed at least as well as SHFM in predicting cumulative mortality, but any model that included the SHFM appeared to reduce performance according to the proportional hazards model. In contrast, the addition of LCM A and B to SHFM Score and bucindolol treatment improved prediction of one-year mortality according to logistic regression. The c-index for predicting one-year mortality increased from 0.71 to 0.75 with the addition of LCM A (p = 0.02) and to 0.76 with the addition of LCM A and B (p<0.01). The NRI also showed a significant improvement in prediction of one-year mortality with the addition of LCM A and LCM A + B to SHFM and bucindolol treatment (p<0.01 for both). Adding both LCM A and B significantly increased the c-index for predicting LVEF response to 0.71 (p<0.01). The NRI showed statistically significant improvement with addition of LCM A and B individually as well as together (p<0.01 for all). NRI transition matrices are found in the Appendix S1 (Table S3).

**Table 5:** C-indices for predicting outcomes, BEST and validation sets (BEST LOOCV, MOCHA).

	Cumulative mortality			1-Year Mortality			EF Response		
Models	BEST	BEST LOOCV	MOCHA	BEST	BEST LOOCV	MOCHA	BEST	BEST LOOCV	MOCHA
SHFM	0.762	0.749	0.500	0.713	0.703	0.819	0.689	0.684	0.674
LCM A	0.821	0.808	0.743	0.682	0.623	0.544	0.666	0.614	0.684
LCM B	0.804	0.792	0.655	0.669	0.613	0.642	0.679	0.620	0.747
A+B	0.755	0.739	0.703	0.735	0.691	0.596	0.701	0.677	0.761
SHFM+A	0.732	0.720	0.500	0.747	0.726	0.791	0.704	0.691	0.711
SHFM+B	0.753	0.741	0.500	0.720	0.696	0.810	0.700	0.689	0.714
All	0.723	0.713	0.529	0.756	0.725	0.766	0.713	0.695	0.733

### Validation

Leave-one-out-cross-validation and external validation were then performed to verify association between latent class membership and outcomes as well as the added value of combining latent class models with the SHFM. As expected, c-indices for all models for all outcomes were slightly lower using leave-one-out cross-validation (Table 5). The added value of LCM A alone and the combination of LCM A and B to SHFM and treatment group in predicting one-year mortality and LVEF response were redemonstrated, as was the decrease in performance of predicting cumulative all-cause mortality with combined LCM and SHFM models. The LCM A and B subgroup definitions were then used to classify the 166 nonischemic HFREF patients enrolled in MOCHA. Demographics of MOCHA patients according to the components of the latent class models can be found in Tables S4 and S5 of the Appendix S1. One-year mortality and LVEF response were similar in nonischemic patients enrolled in MOCHA and BEST (Appendix S1, Table S6). Survival models that included SHFM did not perform well in MOCHA, though the low number of deaths (6) and a much shorter follow-up time (median = 6 months) likely contributed to the low predictive ability for cumulative mortality using the proportional hazards model. In contrast, models that included SHFM had somewhat better predictive ability for the one-year mortality outcome. Finally, the c-index for predicting LVEF response in the validation dataset increased using SHFM Score and bucindolol alone (c = 0.67) with the addition of LCM A (c = 0.71), B (c = 0.71), and both A and B (c = 0.73).

## Discussion

**Figure 4:**
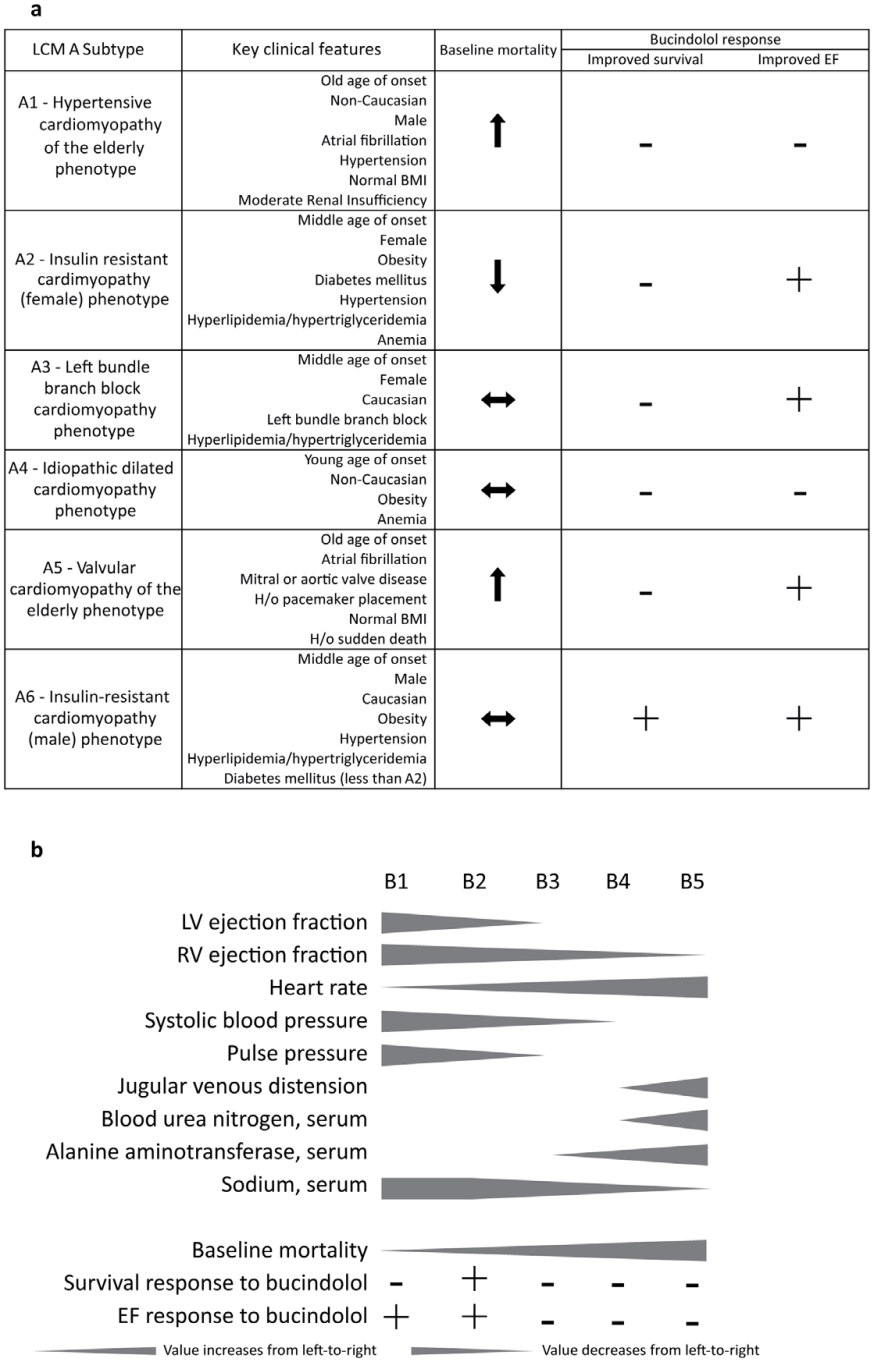
Figure 4a: Key features of Latent Class Model A, mortality trends, and response to bucindolol. Figure 4b: Key features of Latent Class Model B, mortality trends, and response to bucindolol.

Using the combination of high-dimensional clinical phenotyping and latent class analysis, we have identified a number of HFREF subtypes with distinct clinical profiles that demonstrate significant variation in prognosis as measured by all-cause mortality and response to bucindolol as measured by reduction in mortality and increased likelihood of LVEF response (Figure 4). Several of the LCM A subtypes resemble previously described nonischemic HFREF phenotypes, while LCM B subtypes model HF progression and severity. The latent class models, particularly LCM A, remained significantly associated with certain outcomes after combining them with the SHFM, suggesting that the information in the latent class models is different from the information in the SHFM Score. Taken together, these results suggest that our approach to HFREF subtype identification may be useful for identifying patients with potentially ‘reversible’ HFREF as well as those more likely to benefit from bucindolol.

### Insight into Mechanisms of Disease and Treatment Response

Regression models often provide only limited insight into the interactions between clinical processes in a given individual or the importance of a specific process in different contexts. The features of the subtypes identified in the present analysis suggest our approach identifies cohorts of patients who may share underlying pathophysiology and disease prognosis. Defining clinical features of LCM A subtypes, corresponding outcomes, and treatment response are summarized in Figure 4a. Subtypes A1 and A5, both characterized by older age, are notable for high rates of hypertension and valvular disease respectively, which have been identified as two major risk factors for nonischemic HFREF in the elderly. [19] The development of HFREF in the setting of these risk factors has been speculated to be in part due to accumulated DNA damage and telomere attrition, which affect cellular function and apoptosis. [20] These two subtypes also had the highest event rates in LCM A, as might be expected in cardiomyopathy of the elderly. Subtypes A2 and A6 are distinguished by obesity, hypertension, hyperlipidemia, hypertriglyceridemia and diabetes mellitus, which are all characteristics of the metabolic syndrome, [21] and these subtypes may therefore represent variants of insulin-resistant cardiomyopathy. [22] Of note, subtype A2 had the lowest cumulative and one-year mortality and showed no reduction in mortality with bucindolol, while subtype A6, the largest LCM A subtype, was the only LCM A subtype to show a decrease in all-cause mortality. Subtype A3 patients are characterized primarily by the presence of LBBB, a known risk factor for HFREF. LBBB may affect neurohormonal activation and promote myocyte hypertrophy and LV dilation as consequences of ventricular dysynchrony. [23] While some features of the LCM A subtypes are familiar, novel associations were observed such as the predominance of white women and the high prevalence of dyslipidemia in subtype A3, which was characterized primarily by LBBB. Such observations may provide new insight into the mechanisms involved in the pathogenesis of HFREF and suggest hypotheses for future study. In addition, subtypes such as A6 that demonstrated a favorable response to bucindolol even when the overall effect of bucindolol was not significant might be selected for further study regarding the specific role of bucindolol in treatment of HFREF in those patients as well as the mechanism of treatment response.

Trends of key features of the LCM B subtypes are represented in Figure 4b. Specifically, LCM B subtypes with worse prognoses were associated with first with worsening LV and RV ejection fraction followed by evidence of worsening cardiac output (higher heart rate, lower systolic and pulse pressure, and hyponatremia) and finally worsening evidence of volume overload (increasing jugular venous distension, blood urea nitrogen, and alanine aminotransferase). Each of these trends has been associated with poor prognosis in HFREF previously [24]–[33], and even in the analyses that have characterized several markers of severity simultaneously, there has been little quantitative insight gained into the patterns or order in which worsening prognostic signs may appear. [43], [48] In addition, treatment benefit with bucindolol for both all-cause mortality and LVEF response was confined to LCM B stages with lower baseline mortality, higher LV and RVEF, more favorable hemodynamics, and few signs of volume overload. Results of analyses with the SHFM, which uses similar variables (i.e. gender, 100/serum cholesterol, 100/LVEF, systolic blood pressure/10, age/10, 138-serum sodium, |16-serum hemoglobin|), also suggested higher likelihood of LVEF response associated with lower SHFM scores, but the LCM A and B models provide concrete clinical profiles to associate with treatment response.

### Identification of HFREF Subtypes Using Latent Class Analysis

This analysis demonstrates the potential utility of combining high-dimensional clinical phenotyping and latent class analysis for identifying relevant subtypes of HFREF. It is impossible to determine multivariate odds ratios for all of the variables included in the latent class models presented here using a traditional regression model, as the number of possible interactions (26,542,080 and 432,000,000 for LCM A and LCM B, respectively) prevents calculation using realistic sample sizes. Latent class analysis provides a quantitative mechanism of reducing the number of comparisons by aggregating individuals with similar clinical profiles. Our approach produces data-driven definitions of HFREF subtypes that integrate a large number of clinical features but are not dependent on any one feature for classification. Consequently, a feature like age may not have the same implications among all individuals. For example, subtype A4 is associated with worse outcomes than subtypes A2 or A6 despite younger age and lower burden of comorbid diseases. Clinical features may therefore be associated with a conditional probability for different outcomes depending on their context, capturing relevant interactions between comorbid conditions without direct calculation of all possible interactions. The added value of LCM A and B membership to SHFM for predicting survival despite sharing several common variables suggests that LCM A and B subtype may provide additional prognostic information to the SHFM Score. Finally, the variability in clinical outcomes observed between subtypes suggests that this approach could be useful in identifying patients with higher likelihood of HFREF reversibility in the absence of an obvious reversible etiology or conversely for identifying high risk patients for accelerated advanced HFREF therapy.

### Implementation and Sharing

Latent class analysis produces a formal mechanism for classifying any individual patient according to the subtypes presented in this analysis. While manual calculation is possible (Appendix S1), an electronic implementation is more convenient. This could be accomplished using a stand-alone web-based application or by incorporation into an electronic health record system, which will be the subject of future work. Furthermore, it may be possible to develop simplified criteria for identifying LCM A and B types using methods such as classification and/or regression trees to simplify clinical application and validation in other data sets.

### Limitations

This is a retrospective analysis that may not reflect current management of HFREF, and conclusions regarding the prognostic implications of LCM A and B membership must be validated further. We present two methods of validation that suggest that the performance of this model is reproducible, but the number nonischemic patients in MOCHA was relatively small with only six deaths during the one year of follow-up, and a range of dosing was used in the carvedilol treatment arms, which both likely affected validation of mortality outcomes. Carvedilol is also known to be a more effective β-blocker on a population level, which could also affect performance estimates in the validation set. Specifically, carvedilol has been shown to have a stronger mortality benefit than bucindolol on a population level and is known to be associated with marked improvement in LVEF. [13], [49]–[53] These differences compared with bucindolol may explain why the performance of the LCM models in the MOCHA trial appeared worse in predicting mortality and better in predicting LVEF response, as they were calibrated using bucindolol. Furthermore, LCM performance could be assessed in patients enrolled in MOCHA collectively, but the numbers for each subtype in MOCHA, particularly with respect to the placebo arm, were insufficient to validate subtype-specific outcomes. Additional validation with larger populations is therefore necessary before membership in one of the subtypes presented here could be used to tailor HFREF therapy.

There were marked discrepancies between the performance assessments of the combinations of LCM A and B models and the SHFM using the proportional hazards model and logistic regression for predicting mortality. The SHFM did not perform as well towards predicting cumulative survival, whereas it showed a slight advantage over the LCM models for the one-year outcomes. In addition, there were discrepancies between relative performance of SHFM in BEST and MOCHA. The SHFM c-index for MOCHA according to the proportional hazards model was far lower than has been described in any other validation set, although the c-index using the logistic regression model was comparable to previous assessments and its performance in BEST. [43] This may be related to the low number of events and short duration of follow-up in MOCHA as discussed earlier or to differences in methodology for estimating the c-index for the respective survival functions.

Another important limitation is the generalizability of these latent class definitions. Utilization of the coefficients derived in this analysis to determine LCM subtype for other patients assumes that the patient population is the same as the nonischemic patients enrolled in BEST. This assumption may be particularly problematic for LCM B, which includes LVEF in its definition. Like all clinical trials, the inclusion and exclusion criteria of the BEST study are a critical source of selection bias and limit the generalizability of any predictive models developed from BEST to patients that do not meet those entry criteria. [14] This is especially relevant for data-driven latent class models like those presented here, as subtype definitions are by definition dependent on the original study population, and patient subtypes not present in the derivation population might be misidentified. It must also be remembered that latent classes only represent patterns of the variables included in the models, and that those latent classes may not necessarily exist as recognizable patient types in an independent population, [6] due in part to other variables that may be important in a disease process. The utility of these models must therefore be validated further in other patient populations, and the definitions of subtypes will need to be revised over time as more diverse patient populations are incorporated.

Latent class analysis is helpful in identifying highly prevalent subtypes of patients. However, subtypes with only a small number of individuals may be missed. This can be addressed by increasing the hypothesized number of subtypes in each model to identify poorly represented subtypes, but the danger of over-fitting increases with the number of latent classes and must be balanced with the goal of identifying more subtypes of disease.

### Conclusion

High-dimensional phenotyping combined with latent class analysis provide a method of identifying subtypes of nonischemic HFREF patients who may have shared pathophysiology with implications for prognosis and response to bucindolol therapy. Significant reduction in all-cause mortality and increase in likelihood of LVEF response was associated with bucindolol treatment in specific groups identified using these classification methods. Identification of patients’ HFREF subtype may provide a means of personalizing clinical prognosis and estimating likelihood of responding to medical treatment.

## Supporting Information

Appendix S1 (DOCX)
