# Supplementary material for: A Personalized BEST: Characterization of Latent Clinical Classes of Nonischemic Heart Failure That Predict Outcomes and Response to Bucindolol
Source: PLoS One. 2012 Nov 7;7(11):e48184. doi: 10.1371/journal.pone.0048184 (PMC3492337; doi:10.1371/journal.pone.0048184)
Supplement: Appendix S1 — (DOCX) [file pone.0048184.s001.docx]

**Appendix**

**Recruitment Sites**

Albany Medical Center; Albert Einstein College of Medicine; Baptist Memorial Hospital; Baylor College of Medicine; Cardiology of Tulsa; Cedars-Sinai Medical Center; Cleveland Clinic Foundation; Cook County Hospital; Dartmouth Hitchcock Medical Center; Duke University Medical Center; Elmhurst Hospital Center; George Washington University; Georgetown University Hospital; Grady Memorial Hospital; Heart Care Midwest; Hospital of the University of Pennsylvania; Johns Hopkins Hospital; LDS Hospital; Louisiana State University; Loyola University Medical Center; Maricopa Medical Research Foundation; Mayo Clinic, Rochester; Mayo Clinic, Scottsdale; MCP Hahnemann University; Medical College of Virginia; Medical College of Wisconsin; Medical University of South Carolina; Minneapolis Heart Institute; Montreal Heart Institute; Morristown Memorial Hospital; National Naval Medical Center; Nebraska Heart Institute; New England Medical Center; Oklahoma Foundation for Cardiology Research; Oregon Health Sciences University; New Mexico Heart Institute; Pennsylvania State University Hospital; Robert Wood Johnson Medical School; Shands Hospital, University of Florida; St. John’s Mercy Medical Center; University of Texas Southwestern; Washington University School of Medicine; University of Alabama Medical Center; University of California, San Diego, Medical Center; University of Cincinnati; University of Colorado Health Science Center; University of Connecticut Health Center; University of Florida Health Science Center; University of Iowa Hospital; University of Maryland; University Medical Center, University of Arizona; University of Minnesota Hospital; University of Mississippi Medical Center; University of Montreal; University of North Carolina; University of Pittsburgh Medical Center; University of Rochester Medical Center; University of Utah Health Science Center; University of Wisconsin Hospital and Clinics; Watson Clinic; Yale-New Haven Hospital; Veterans Affairs Medical Center (VAMC) Baltimore; VAMC Boston; VAMC Bronx; VAMC Charleston; VAMC Dallas; VAMC Denver; VAMC Durham; VAMC Fresno; VAMC Hines; VAMC Houston; VAMC Jackson; VAMC Lexington; VAMC Little Rock; VAMC Long Beach; VAMC Madison; VAMC Memphis; VAMC Minneapolis; VAMC Newington; VAMC Portland; VAMC Richmond; VAMC St. Louis; VAMC Salem; VAMC San Francisco; VAMC San Diego; VAMC Sepulveda; VAMC San Antonio; VAMC Tampa; VAMC Tucson; and VAMC Washington, D.C

Example calculation for LCM A:

White woman with HF onset at age 51, BMI 28 kg/m^2, creatinine clearance of 55 ml/min*1.73 m2, hyperlipidemia with a total cholesterol of 210 mg/dL, hypertriglyceridemia with serum triglycerides = 240 mg/dL, hypertension with a blood pressure of 150/90 mm Hg, left bundle branch block, and a hematocrit of 37%. The following are Bayesian partial probability calculations using coefficients from Appendix Table B:

*A1*:

0.186 (population share)*

0.376 (age=51) *0.177 (female)*

0.381 (white) *

0.193 (BMI = 28)*

0.739 (no diabetes) *

0.643 (BP 150/90) *

0.194 (cholesterol = 210 mg/dL) *

0.177 (triglycerides = 240 mg/dL) *

0.504 (creatinine clearance = 55) *

0.556 (hematocrit = 37%) *

0.747 (no atrial fibrillation) *

0.227 (left bundle branch block present) *

0.924 (no pacemaker) *

0.971 (no mitral valve disease) *

0.960 (no aortic valve disease) *

0.968 (no history of sudden cardiac death) = **3.83502*10^-7^**

*A2* (category coefficients in same order as for A1): 0.144*0.404*0.551*0.464*0.326*0.305*0.583*0.164*0.217*0.511*0.376*0.891*

0.199*1*1*1*0.955 = **9.98265*10^-7^**

*A3*: 6.18331*10^-5^

*A4*: 1.41343*10^-7^

*A5*: 1.12669*10^-7^

*A6*: 0

Sum of partial probabilities for LCM A 1-6 = 3.83*10^-7^+9.98*10^-7^+6.18*10^-5^+1.41*10^-7^+1.13*10^-7^+0 = 6.35*10^-5^

Final probability of class membership:

A1: 3.83*10^-7^/6.35*10^-5^ = 0.0060

A2: 9.98*10^-7^/6.35*10^-5^ = 0.016

**A3: 6.183*10^-5^/6.35*10^-5^ = 0.974 -> patient classified as A3**

A4: 1.41*10^-7^/6.35*10^-5^ = 0.0022

A5: 1.13*10^-7^/6.35*10^-5^ = 0.0018

A6: 0/6.35*10^-5^ = 0

**Table S1 – Class conditional probabilities for latent class model A**

|  | A1 | A2 | A3 | A4 | A5 | A6 |
| --- | --- | --- | --- | --- | --- | --- |
|  | 18.6% (208) | 14.4% (161) | 16.6% (186) | 14.5% (162) | 7.8% (87) | 28.3% (317) |
| Age of HF onset, years |  |  |  |  |  |  |
| < 30 | 0.000 | 0.033 | 0.000 | 0.336 | 0.015 | 0.021 |
| 30-45 | 0.154 | 0.251 | 0.182 | 0.540 | 0.118 | 0.299 |
| 45-60 | 0.376 | 0.404 | 0.519 | 0.124 | 0.376 | 0.495 |
| > 60 | 0.470 | 0.312 | 0.300 | 0.000 | 0.491 | 0.184 |
| Gender |  |  |  |  |  |  |
| Male | 0.823 | 0.449 | 0.342 | 0.502 | 0.782 | 1.000 |
| Female | 0.177 | 0.551 | 0.658 | 0.498 | 0.218 | 0.000 |
| Race |  |  |  |  |  |  |
| White, non-Hispanic | 0.381 | 0.464 | 0.942 | 0.343 | 0.854 | 0.677 |
| Black, non-Hispanic | 0.529 | 0.442 | 0.025 | 0.545 | 0.080 | 0.242 |
| Hispanic | 0.072 | 0.077 | 0.034 | 0.113 | 0.021 | 0.053 |
| Asian/Pacific Islander | 0.018 | 0.006 | 0.000 | 0.000 | 0.024 | 0.010 |
| American Indian | 0.000 | 0.011 | 0.000 | 0.000 | 0.000 | 0.014 |
| Other | 0.000 | 0.000 | 0.000 | 0.000 | 0.022 | 0.003 |
| Body Mass Index, kg/m^2^ |  |  |  |  |  |  |
| < 18.5 | 0.078 | 0.000 | 0.028 | 0.000 | 0.030 | 0.000 |
| 18.5-25 | 0.624 | 0.179 | 0.319 | 0.258 | 0.496 | 0.119 |
| 25-30 | 0.193 | 0.326 | 0.316 | 0.238 | 0.370 | 0.368 |
| > 30 | 0.105 | 0.495 | 0.338 | 0.505 | 0.104 | 0.514 |
| Diabetes Mellitus |  |  |  |  |  |  |
| None | 0.739 | 0.305 | 0.846 | 0.906 | 0.930 | 0.687 |
| Present | 0.203 | 0.481 | 0.096 | 0.088 | 0.047 | 0.227 |
| Present with end-organ damage | 0.058 | 0.214 | 0.058 | 0.006 | 0.023 | 0.086 |
| Hypertension |  |  |  |  |  |  |
| None | 0.158 | 0.000 | 0.145 | 0.173 | 0.282 | 0.011 |
| Borderline | 0.114 | 0.064 | 0.411 | 0.231 | 0.343 | 0.178 |
| Present | 0.634 | 0.583 | 0.418 | 0.428 | 0.295 | 0.589 |
| Severe | 0.093 | 0.353 | 0.026 | 0.168 | 0.080 | 0.221 |
| Total cholesterol, mg/dL |  |  |  |  |  |  |
| < 200 | 0.642 | 0.069 | 0.128 | 0.576 | 0.420 | 0.262 |
| 200-240 | 0.194 | 0.164 | 0.272 | 0.265 | 0.295 | 0.224 |
| > 240 | 0.164 | 0.766 | 0.600 | 0.159 | 0.285 | 0.514 |
| Triglycerides, mg/dL |  |  |  |  |  |  |
| < 150 | 0.883 | 0.061 | 0.109 | 0.450 | 0.399 | 0.050 |
| 150-250 | 0.117 | 0.217 | 0.335 | 0.364 | 0.321 | 0.386 |
| > 250 | 0.000 | 0.690 | 0.556 | 0.186 | 0.280 | 0.564 |
| Creat. Cl., ml/min*1.73m^2^ |  |  |  |  |  |  |
| > 90 | 0.030 | 0.046 | 0.100 | 0.332 | 0.058 | 0.139 |
| 60-90 | 0.358 | 0.273 | 0.431 | 0.517 | 0.277 | 0.531 |
| 30-60 | 0.504 | 0.511 | 0.436 | 0.152 | 0.581 | 0.301 |
| 15-30 | 0.097 | 0.143 | 0.033 | 0.000 | 0.083 | 0.029 |
| < 15 | 0.011 | 0.026 | 0.000 | 0.000 | 0.000 | 0.000 |
| Hematocrit, % |  |  |  |  |  |  |
| >40 | 0.049 | 0.000 | 0.000 | 0.000 | 0.056 | 0.126 |
| 30-40 | 0.556 | 0.376 | 0.473 | 0.488 | 0.580 | 0.874 |
| 20-30 | 0.366 | 0.608 | 0.527 | 0.492 | 0.364 | 0.000 |
| < 20 | 0.028 | 0.016 | 0.000 | 0.020 | 0.000 | 0.000 |
| Atrial fibrillation |  |  |  |  |  |  |
| Yes | 0.253 | 0.109 | 0.083 | 0.058 | 0.806 | 0.221 |
| No | 0.747 | 0.891 | 0.917 | 0.942 | 0.194 | 0.779 |
| Left bundle branch block |  |  |  |  |  |  |
| Yes | 0.227 | 0.199 | 0.670 | 0.081 | 0.107 | 0.158 |
| No | 0.773 | 0.801 | 0.330 | 0.919 | 0.893 | 0.842 |
| Pacemaker |  |  |  |  |  |  |
| Yes | 0.076 | 0.000 | 0.006 | 0.043 | 0.379 | 0.036 |
| No | 0.924 | 1.000 | 0.994 | 0.957 | 0.621 | 0.964 |
| Mitral valve disease |  |  |  |  |  |  |
| Yes | 0.029 | 0.000 | 0.032 | 0.014 | 0.430 | 0.021 |
| No | 0.971 | 1.000 | 0.968 | 0.986 | 0.570 | 0.979 |
| Aortic valve disease |  |  |  |  |  |  |
| Yes | 0.040 | 0.000 | 0.014 | 0.000 | 0.201 | 0.011 |
| No | 0.960 | 1.000 | 0.986 | 1.000 | 0.799 | 0.989 |
| History of sudden cardiac death |  |  |  |  |  |  |
| Yes | 0.032 | 0.045 | 0.054 | 0.032 | 0.133 | 0.021 |
| No | 0.968 | 0.955 | 0.946 | 0.968 | 0.867 | 0.979 |

**Table S2 – Class conditional probabilities for latent class model B**

|  | B1 | B2 | B3 | B4 | B5 |
| --- | --- | --- | --- | --- | --- |
| Population share | 22.9%  (247) | 34.1%  (368) | 22.4%  (242) | 11.6%  (125) | 9.1%  (98) |
| Age, years |  |  |  |  |  |
| < 30 | 0.000 | 0.035 | 0.000 | 0.208 | 0.057 |
| 30-45 | 0.009 | 0.293 | 0.073 | 0.507 | 0.197 |
| 45-60 | 0.269 | 0.487 | 0.425 | 0.267 | 0.385 |
| > 60 | 0.727 | 0.185 | 0.502 | 0.018 | 0.361 |
| LVEF, % |  |  |  |  |  |
| > 55 | 0.004 | 0.000 | 0.000 | 0.000 | 0.000 |
| 45-55 | 0.000 | 0.000 | 0.000 | 0.000 | 0.000 |
| 35-45 | 0.008 | 0.003 | 0.000 | 0.000 | 0.000 |
| 25-35 | 0.584 | 0.493 | 0.211 | 0.351 | 0.139 |
| < 25 | 0.405 | 0.504 | 0.789 | 0.649 | 0.861 |
| RVEF % |  |  |  |  |  |
| > 55 | 0.209 | 0.071 | 0.065 | 0.059 | 0.000 |
| 45-55 | 0.208 | 0.185 | 0.095 | 0.129 | 0.080 |
| 35-45 | 0.275 | 0.296 | 0.188 | 0.244 | 0.247 |
| 25-35 | 0.193 | 0.267 | 0.315 | 0.227 | 0.151 |
| < 25 | 0.115 | 0.181 | 0.338 | 0.342 | 0.522 |
| QRS, msec |  |  |  |  |  |
| < 120 | 0.389 | 0.756 | 0.453 | 0.735 | 0.574 |
| 120-150 | 0.235 | 0.066 | 0.189 | 0.082 | 0.214 |
| > 150 | 0.376 | 0.179 | 0.358 | 0.183 | 0.213 |
| Heart rate, bpm |  |  |  |  |  |
| < 60 | 0.131 | 0.051 | 0.061 | 0.042 | 0.054 |
| 60-80 | 0.541 | 0.370 | 0.367 | 0.233 | 0.190 |
| 80-100 | 0.310 | 0.419 | 0.492 | 0.469 | 0.499 |
| 100-120 | 0.019 | 0.147 | 0.074 | 0.197 | 0.227 |
| > 120 | 0.000 | 0.013 | 0.007 | 0.059 | 0.031 |
| Systolic blood pressure, mm Hg |  |  |  |  |  |
| >120 | 0.698 | 0.664 | 0.079 | 0.000 | 0.229 |
| 110-120 | 0.203 | 0.267 | 0.158 | 0.064 | 0.108 |
| 100-110 | 0.086 | 0.066 | 0.299 | 0.401 | 0.191 |
| 90-110 | 0.013 | 0.003 | 0.306 | 0.421 | 0.256 |
| <90 | 0.000 | 0.000 | 0.158 | 0.115 | 0.215 |
| Pulse pressure, mm HG |  |  |  |  |  |
| > 40 | 0.909 | 0.670 | 0.103 | 0.132 | 0.267 |
| 25-40 | 0.091 | 0.319 | 0.773 | 0.730 | 0.511 |
| < 25 | 0.000 | 0.012 | 0.123 | 0.137 | 0.222 |
| Jugular venous distension |  |  |  |  |  |
| Not present | 0.603 | 0.632 | 0.527 | 0.555 | 0.292 |
| Base of neck | 0.281 | 0.235 | 0.293 | 0.237 | 0.230 |
| Halfway up | 0.088 | 0.108 | 0.139 | 0.151 | 0.319 |
| Angle of mandible | 0.028 | 0.025 | 0.041 | 0.057 | 0.159 |
| Blood Urea Nitrogen, mg/dL |  |  |  |  |  |
| < 10 | 0.018 | 0.174 | 0.025 | 0.163 | 0.000 |
| 10-25 | 0.679 | 0.777 | 0.679 | 0.794 | 0.082 |
| 25-40 | 0.192 | 0.048 | 0.250 | 0.011 | 0.388 |
| 40-55 | 0.071 | 0.000 | 0.046 | 0.031 | 0.217 |
| > 55 | 0.040 | 0.000 | 0.000 | 0.313 | 0.779 |
| Alanine aminotransferase, U/L |  |  |  |  |  |
| < 25 | 0.779 | 0.464 | 0.598 | 0.377 | 0.535 |
| 25-50 | 0.206 | 0.442 | 0.320 | 0.510 | 0.265 |
| 50-75 | 0.015 | 0.072 | 0.073 | 0.080 | 0.131 |
| > 75 | 0.000 | 0.022 | 0.010 | 0.034 | 0.068 |
| Serum sodium, mEq/L |  |  |  |  |  |
| > 140 | 0.378 | 0.346 | 0.301 | 0.172 | 0.138 |
| 130-140 | 0.610 | 0.650 | 0.690 | 0.802 | 0.799 |
| < 130 | 0.012 | 0.004 | 0.009 | 0.026 | 0.062 |
| Body Mass Index, kg/m^2^ |  |  |  |  |  |
| < 18.5 | 0.034 | 0.005 | 0.053 | 0.005 | 0.000 |
| 18.5-25 | 0.403 | 0.153 | 0.425 | 0.288 | 0.365 |
| 25-30 | 0.386 | 0.249 | 0.332 | 0.313 | 0.197 |
| > 30 | 0.177 | 0.594 | 0.191 | 0.394 | 0.438 |
| Creat. Clearance, ml/min*1.73m^2^ |  |  |  |  |  |
| > 90 | 0.021 | 0.204 | 0.000 | 0.383 | 0.000 |
| 60-90 | 0.278 | 0.611 | 0.365 | 0.549 | 0.050 |
| 30-60 | 0.576 | 0.180 | 0.635 | 0.061 | 0.059 |
| 15-30 | 0.110 | 0.005 | 0.000 | 0.000 | 0.342 |
| < 15 | 0.015 | 0.000 | 0.000 | 0.008 | 0.018 |
| Hematocrit, % |  |  |  |  |  |
| >40 | 0.011 | 0.032 | 0.064 | 0.068 | 0.101 |
| 30-40 | 0.457 | 0.710 | 0.625 | 0.531 | 0.443 |
| 20-30 | 0.521 | 0.258 | 0.307 | 0.370 | 0.422 |
| < 20 | 0.010 | 0.000 | 0.005 | 0.031 | 0.035 |

**Table S3 – Reclassification matrices when adding LCM A, LCM B and both LCM A and B to SHFM + treatment group.**

| Treatment + SFHM  + A |  | Alive at 1 yr | Dead at 1 yr | No EF response | EF response |
| --- | --- | --- | --- | --- | --- |
|  | Prob ↓ | 593 (59%) | 42 (39%) | 195 (67%) | 415 (50%) |
|  | Prob ↑ | 420 (41%) | 66 (61%) | 98 (33%) | 413 (50%) |
|  | Total | 1013 | 108 | 293 | 828 |
|  |  |  |  |  |  |
| Treatment + SFHM  + B |  | Alive at 1 yr | Dead at 1 yr | No EF response | EF response |
|  | Prob ↓ | 610 (60%) | 62 (57%) | 173 (59%) | 401 (48%) |
|  | Prob ↑ | 403 (40%) | 46 (43%) | 120 (41%) | 427 (52%) |
|  | Total | 1013 | 108 | 293 | 828 |
|  |  |  |  |  |  |
| Treatment + SFHM  + A  + B |  | Alive at 1 yr | Dead at 1 yr | No EF response | EF response |
|  | Prob ↓ | 598 (59%) | 38 (35%) | 184 (63%) | 363 (44%) |
|  | Prob ↑ | 415 (41%) | 70 (65%) | 109 (37%) | 465 (56%) |
|  | Total | 1013 | 108 | 293 | 828 |

*Improved performance is implied by higher percentages in grey-shaded boxes and lower percentages in non-shaded boxes

**Table S4 – Demographics of patients enrolled in MOCHA according to LCM A classification**

|  | A1 | A2 | A3 | A4 | A5 | A6 | All |
| --- | --- | --- | --- | --- | --- | --- | --- |
|  | 13.3% (22) | 6.0% (10) | 22.3% (37) | 8.4% (14) | 23.5% (39) | 26.5% (44) | n=166 |
| Age of HF onset, years |  |  |  |  |  |  |  |
| < 30 | 0.0% | 0.0% | 0.0% | 50.0% | 5.1% | 4.5% | 6.6% |
| 30-45 | 22.7% | 50.0% | 18.9% | 42.9% | 17.9% | 31.8% | 26.5% |
| 45-60 | 31.8% | 30.0% | 54.1% | 7.1% | 41.0% | 56.8% | 43.4% |
| > 60 | 45.5% | 20.0% | 24.3% | 0.0% | 35.9% | 6.8% | 22.9% |
| Male | 86.4% | 30.0% | 16.2% | 42.9% | 82.1% | 100.0% | 66.3% |
| Race |  |  |  |  |  |  |  |
| White, non-Hispanic | 50.0% | 40.0% | 100.0% | 28.6% | 82.1% | 72.7% | 72.3% |
| Black, non-Hispanic | 50.0% | 60.0% | 0.0% | 71.4% | 5.1% | 20.5% | 22.9% |
| Other | 0.0% | 0.0% | 0.0% | 0.0% | 12.8% | 6.8% | 4.8% |
| Body Mass Index, kg/m2 |  |  |  |  |  |  |  |
| < 18.5 | 9.1% | 0.0% | 8.1% | 0.0% | 0.0% | 0.0% | 3.0% |
| 18.5-25 | 59.1% | 0.0% | 45.9% | 7.1% | 53.8% | 2.3% | 31.9% |
| 25-30 | 31.8% | 70.0% | 32.4% | 35.7% | 33.3% | 45.5% | 38.6% |
| > 30 | 0.0% | 30.0% | 13.5% | 57.1% | 10.3% | 52.3% | 25.9% |
| Diabetes Mellitus |  |  |  |  |  |  |  |
| None | 59.1% | 40.0% | 86.5% | 85.7% | 92.3% | 63.6% | 75.3% |
| Present | 31.8% | 50.0% | 13.5% | 14.3% | 2.6% | 36.4% | 21.7% |
| Present with end-organ damage | 9.1% | 10.0% | 0.0% | 0.0% | 5.1% | 0.0% | 3.0% |
| Blood pressure, mm Hg |  |  |  |  |  |  |  |
| < 120/80 | 54.5% | 0.0% | 40.5% | 42.9% | 56.4% | 9.1% | 35.5% |
| 120-140/80-90 | 13.6% | 10.0% | 51.4% | 28.6% | 28.2% | 54.5% | 37.3% |
| 140-160/90-100 | 22.7% | 60.0% | 8.1% | 28.6% | 7.7% | 31.8% | 21.1% |
| > 160/100 | 9.1% | 30.0% | 0.0% | 0.0% | 7.7% | 4.5% | 6.0% |
| Total cholesterol, mg/dL |  |  |  |  |  |  |  |
| < 200 | 68.2% | 0.0% | 21.6% | 50.0% | 48.7% | 22.7% | 35.5% |
| 200-240 | 18.2% | 20.0% | 37.8% | 35.7% | 28.2% | 20.5% | 27.1% |
| > 240 | 13.6% | 80.0% | 40.5% | 14.3% | 23.1% | 56.8% | 37.3% |
| Creat. Cl., ml/min*1.73m2 |  |  |  |  |  |  |  |
| > 90 | 0.0% | 10.0% | 0.0% | 0.0% | 0.0% | 4.5% | 1.8% |
| 60-90 | 9.1% | 40.0% | 40.5% | 71.4% | 30.8% | 40.9% | 36.7% |
| 30-60 | 68.2% | 40.0% | 56.8% | 28.6% | 64.1% | 54.5% | 56.0% |
| 15-30 | 22.7% | 10.0% | 2.7% | 0.0% | 5.1% | 0.0% | 5.4% |
| < 15 | 0.0% | 0.0% | 0.0% | 0.0% | 0.0% | 0.0% | 0.0% |
| Hematocrit, % |  |  |  |  |  |  |  |
| >40 | 18.2% | 0.0% | 0.0% | 0.0% | 7.7% | 11.4% | 7.2% |
| 30-40 | 40.9% | 40.0% | 51.4% | 42.9% | 66.7% | 88.6% | 62.0% |
| 20-30 | 40.9% | 60.0% | 48.6% | 57.1% | 25.6% | 0.0% | 30.7% |
| < 20 | 0.0% | 0.0% | 0.0% | 0.0% | 0.0% | 0.0% | 0.0% |
| Atrial fibrillation | 13.6% | 0.0% | 0.0% | 0.0% | 33.3% | 15.9% | 13.9% |
| Left bundle branch block | 4.5% | 0.0% | 24.3% | 0.0% | 2.6% | 2.3% | 7.2% |
| Pacemaker | 0.0% | 0.0% | 0.0% | 0.0% | 12.8% | 0.0% | 3.0% |
| Mitral valve disease | 0.0% | 0.0% | 10.8% | 7.1% | 35.9% | 4.5% | 12.7% |
| Aortic valve disease | 0.0% | 0.0% | 0.0% | 0.0% | 7.7% | 0.0% | 1.8% |
| History of sudden cardiac death | 4.5% | 0.0% | 0.0% | 0.0% | 5.1% | 0.0% | 1.8% |

**Table S5 – Demographics of patients enrolled in MOCHA according to LCM B classification (RVEF not measured)**

|  | B1 | B2 | B3 | B4 | B5 | All subjects |
| --- | --- | --- | --- | --- | --- | --- |
|  | 32.8% (41) | 28.8% (36) | 10.4% (13) | 52% (65) | 8.8% (11) | n=166 |
| Age, years |  |  |  |  |  |  |
| < 30 | 7.3% | 0.0% | 15.4% | 0.0% | 0.0% | 1.6% |
| 30-45 | 34.1% | 0.0% | 69.2% | 9.2% | 18.2% | 13.6% |
| 45-60 | 56.1% | 30.6% | 15.4% | 47.7% | 54.5% | 40.0% |
| > 60 | 2.4% | 69.4% | 0.0% | 43.1% | 27.3% | 44.8% |
| LVEF, % |  |  |  |  |  |  |
| > 55 | 0.0% | 0.0% | 0.0% | 0.0% | 0.0% | 0.0% |
| 45-55 | 0.0% | 0.0% | 0.0% | 0.0% | 0.0% | 0.0% |
| 35-45 | 2.4% | 8.3% | 0.0% | 0.0% | 0.0% | 2.4% |
| 25-35 | 56.1% | 47.2% | 30.8% | 13.8% | 18.2% | 25.6% |
| < 25 | 41.5% | 44.4% | 69.2% | 86.2% | 81.8% | 72.0% |
| QRS, msec |  |  |  |  |  |  |
| < 120 | 85.4% | 38.9% | 92.3% | 49.2% | 54.5% | 51.2% |
| 120-150 | 2.4% | 25.0% | 7.7% | 16.9% | 9.1% | 17.6% |
| > 150 | 12.2% | 36.1% | 0.0% | 33.8% | 36.4% | 31.2% |
| Heart rate, bpm |  |  |  |  |  |  |
| < 60 | 0.0% | 0.0% | 0.0% | 0.0% | 0.0% | 0.0% |
| 60-80 | 31.7% | 50.0% | 38.5% | 36.9% | 45.5% | 41.6% |
| 80-100 | 51.2% | 47.2% | 15.4% | 50.8% | 27.3% | 44.0% |
| 100-120 | 17.1% | 2.8% | 38.5% | 12.3% | 27.3% | 13.6% |
| > 120 | 0.0% | 0.0% | 7.7% | 0.0% | 0.0% | 0.8% |
| Systolic blood pressure, mm Hg |  |  |  |  |  |  |
| >120 | 61.0% | 69.4% | 0.0% | 6.2% | 27.3% | 25.6% |
| 110-120 | 36.6% | 25.0% | 7.7% | 16.9% | 9.1% | 17.6% |
| 100-110 | 2.4% | 5.6% | 46.2% | 38.5% | 18.2% | 28.0% |
| 90-110 | 0.0% | 0.0% | 38.5% | 26.2% | 27.3% | 20.0% |
| <90 | 0.0% | 0.0% | 7.7% | 12.3% | 18.2% | 8.8% |
| Pulse pressure, mm HG |  |  |  |  |  |  |
| > 40 | 70.7% | 100.0% | 0.0% | 6.2% | 27.3% | 34.4% |
| 25-40 | 29.3% | 0.0% | 69.2% | 84.6% | 63.6% | 56.8% |
| < 25 | 0.0% | 0.0% | 30.8% | 9.2% | 9.1% | 8.8% |
| Blood Urea Nitrogen, mg/dL |  |  |  |  |  |  |
| < 10 | 24.4% | 2.8% | 23.1% | 0.0% | 0.0% | 3.2% |
| 10-25 | 75.6% | 63.9% | 76.9% | 73.8% | 9.1% | 65.6% |
| 25-40 | 0.0% | 22.2% | 0.0% | 24.6% | 18.2% | 20.8% |
| 40-55 | 0.0% | 2.8% | 0.0% | 1.5% | 0.0% | 1.6% |
| > 55 | 0.0% | 8.3% | 0.0% | 0.0% | 18.2% | 4.0% |
| Alanine aminotransferase, U/L |  |  |  |  |  |  |
| < 25 | 51.2% | 80.6% | 76.9% | 78.5% | 81.8% | 79.2% |
| 25-50 | 36.6% | 19.4% | 15.4% | 18.5% | 18.2% | 18.4% |
| 50-75 | 7.3% | 0.0% | 0.0% | 3.1% | 0.0% | 1.6% |
| > 75 | 4.9% | 0.0% | 7.7% | 0.0% | 0.0% | 0.8% |
| Serum sodium, mEq/L |  |  |  |  |  |  |
| > 140 | 36.6% | 66.7% | 23.1% | 49.2% | 9.1% | 48.0% |
| 130-140 | 63.4% | 33.3% | 76.9% | 50.8% | 90.9% | 52.0% |
| < 130 | 0.0% | 0.0% | 0.0% | 0.0% | 0.0% | 0.0% |
| Body Mass Index, kg/m2 |  |  |  |  |  |  |
| < 18.5 | 4.9% | 5.6% | 0.0% | 1.5% | 0.0% | 2.4% |
| 18.5-25 | 4.9% | 41.7% | 38.5% | 40.0% | 45.5% | 40.8% |
| 25-30 | 46.3% | 44.4% | 30.8% | 33.8% | 27.3% | 36.0% |
| > 30 | 43.9% | 5.6% | 30.8% | 24.6% | 27.3% | 20.0% |
| Creat. Clearance, ml/min*1.73m2 |  |  |  |  |  |  |
| > 90 | 4.9% | 0.0% | 7.7% | 0.0% | 0.0% | 0.8% |
| 60-90 | 73.2% | 19.4% | 76.9% | 21.5% | 0.0% | 24.8% |
| 30-60 | 22.0% | 72.2% | 15.4% | 78.5% | 45.5% | 67.2% |
| 15-30 | 0.0% | 8.3% | 0.0% | 0.0% | 54.5% | 7.2% |
| < 15 | 0.0% | 0.0% | 0.0% | 0.0% | 0.0% | 0.0% |
| Hematocrit, % |  |  |  |  |  |  |
| >40 | 0.0% | 5.6% | 0.0% | 13.8% | 9.1% | 9.6% |
| 30-40 | 78.0% | 55.6% | 61.5% | 61.5% | 27.3% | 56.8% |
| 20-30 | 22.0% | 38.9% | 38.5% | 24.6% | 63.6% | 33.6% |
| < 20 | 0.0% | 0.0% | 0.0% | 0.0% | 0.0% | 0.0% |

**Table S6 – Outcomes and SHFM Score of patients enrolled in MOCHA according to LCM A and B classification – All mortality events documented occurred < 12 months after enrollment**

| LCM A | Total Number | | Pred 1-yr mortality, SHFM | | Mortality | | EF response | |
| --- | --- | --- | --- | --- | --- | --- | --- | --- |
|  | Placebo | Carvedilol | Plac,% | Carv,% | Plac,% | Carv,% | Plac,% | Carv,% |
| A1 | 6 | 16 | 27.0 | 23.1 | 0.0 | 13.0 | 0.0 | 13.0 |
| A2 | 1 | 9 | 8.7 | 8.2 | 0.0 | 0.0 | 0.0 | 44.0 |
| A3 | 9 | 28 | 19.4 | 11.7 | 22.0 | 0.0 | 0.0 | 32.0 |
| A4 | 3 | 11 | 31.7 | 12.9 | 0.0 | 9.0 | 0.0 | 27.0 |
| A5 | 9 | 30 | 17.7 | 14.5 | 0.0 | 0.0 | 22.0 | 23.0 |
| A6 | 10 | 34 | 13.7 | 10.1 | 20.0 | 6.0 | 20.0 | 44.0 |
| LCM B |  |  |  |  |  |  |  |  |
| B1 | 9 | 32 | 11.5 | 7 | 22.0 | 0.0 | 22.0 | 59.0 |
| B2 | 9 | 27 | 13.0 | 11.6 | 0.0 | 0.0 | 11.0 | 33.0 |
| B3 | 0 | 13 | - | 11.8 | - | 8.0 | - | 31.0 |
| B4 | 18 | 47 | 23.7 | 15.9 | 11.0 | 9.0 | 6.0 | 13.0 |
| B5 | 2 | 9 | 44.4 | 28.3 | 0.0 | 0.0 | 0.0 | 22.0 |
| All | 38 | 128 | 19.3 | 13.2 | 10.5 | 3.9 | 10.5 | 32.0 |
